# CHILDHOOD MALTREATMENT AND PROSOCIAL BEHAVIOR: A QUALITATIVE COMPARATIVE STUDY OF IRISH OLDER ADULT SURVIVORS

**DOI:** 10.1093/geroni/igac059.1253

**Published:** 2022-12-20

**Authors:** Shauna Rohner, Aileen Salas, Alan Carr, Myriam Thoma

**Affiliations:** University of Zürich, Zürich, Zurich, Switzerland; University of Zürich, Zurich, Zurich, Switzerland; University College Dublin, Dublin, Dublin, Ireland; University of Zurich, Zurich, Zurich, Switzerland

## Abstract

Although childhood maltreatment can have lasting effects into later life, positive outcomes have also been observed, including an increased tendency towards prosocial behavior. However, little is known about the link between childhood maltreatment and later life prosocial behavior. Therefore, this study aimed to explore older adult’s experiences of childhood maltreatment and identify mechanisms linked to prosocial behavior in later life. The individual level, but also broader cultural and contextual mechanisms, were considered by comparing two adversity contexts and applying conceptual frameworks (socio-interpersonal framework model of trauma and recovery, motivational process model of altruism born of suffering). Semi-structured interviews (60-120 minutes) were conducted with 29 Irish (older) adult survivors of childhood maltreatment: 17 institutional (welfare care) abuse survivors (mean age: 61 years, range: 50-77), 12 familial abuse survivors (mean age: 58 years, range: 51-72). Interviews were analyzed using Framework Analysis. In both groups at the individual level, enhanced empathy, amelioration, and identity-related mechanisms were linked to prosocial behavior, with connections to caring roles and coping strategies from childhood. On a social contexts level, the limited resources or opportunities for help in childhood, and the social norms and beliefs of that time, influenced participants’ motivation to help others in later life. Group-specific mechanisms were also observed, such as compassion fatigue in the familial sample; and denouncing detrimental societal values in the institutional sample. The identification of individual, adversity-context, and culture-specific mechanisms linked to later-life prosocial behavior can promote a greater understanding of resilience and adaptability in older adult survivors of childhood maltreatment.

